# Supplementary material for: Cell Atlas of The Human Fovea and Peripheral Retina
Source: Sci Rep. 2020 Jun 17;10:9802. doi: 10.1038/s41598-020-66092-9 (PMC7299956; doi:10.1038/s41598-020-66092-9)
Supplement: Supplementary file 1 — Supplementary information. [file 41598_2020_66092_MOESM1_ESM.pdf]

Supplementary information for:

CELL ATLAS OF THE HUMAN FOVEA AND PERIPHERAL RETINA

Wenjun Yan, Yi-Rong Peng, Tavé van Zyl, Aviv Regev, Karthik Shekhar, Dejan Juric, and Joshua R. Sanes

Figure S1

tSNE visualization showing contributions to cell types by batch for photoreceptors (a), horizontal cells (b), bipolar cells (c), amacrine cells (d), retinal ganglion cells (e) and non-neuronal cells (f). Each dot represents one cell. Colors distinguish retins by donor and region (F-fovea, P-peripheral). Source of each sample is shown in Table S1. Because photoreceptors were depleted from peripheral samples, most were obtained from fovea (g). Expression of known photoreceptor type marker genes is plotted in h-l.

Figure S2

Violin and superimposed box plots showing expression of *OPN4* in RGC clusters

Figure S3

Heat maps showing expression levels of disease genes by cell classes in the fovea and periphery. Only genes expressed by more than 20% of cells in any individual class in either fovea or peripheral cells are plotted.

Table S1

Information of donors from whom retinas were obtained.

Table S2

Publications on single cell or single nucleus RNA-seq analyses of human retina.

Table S3

Quality matrix of each collection.

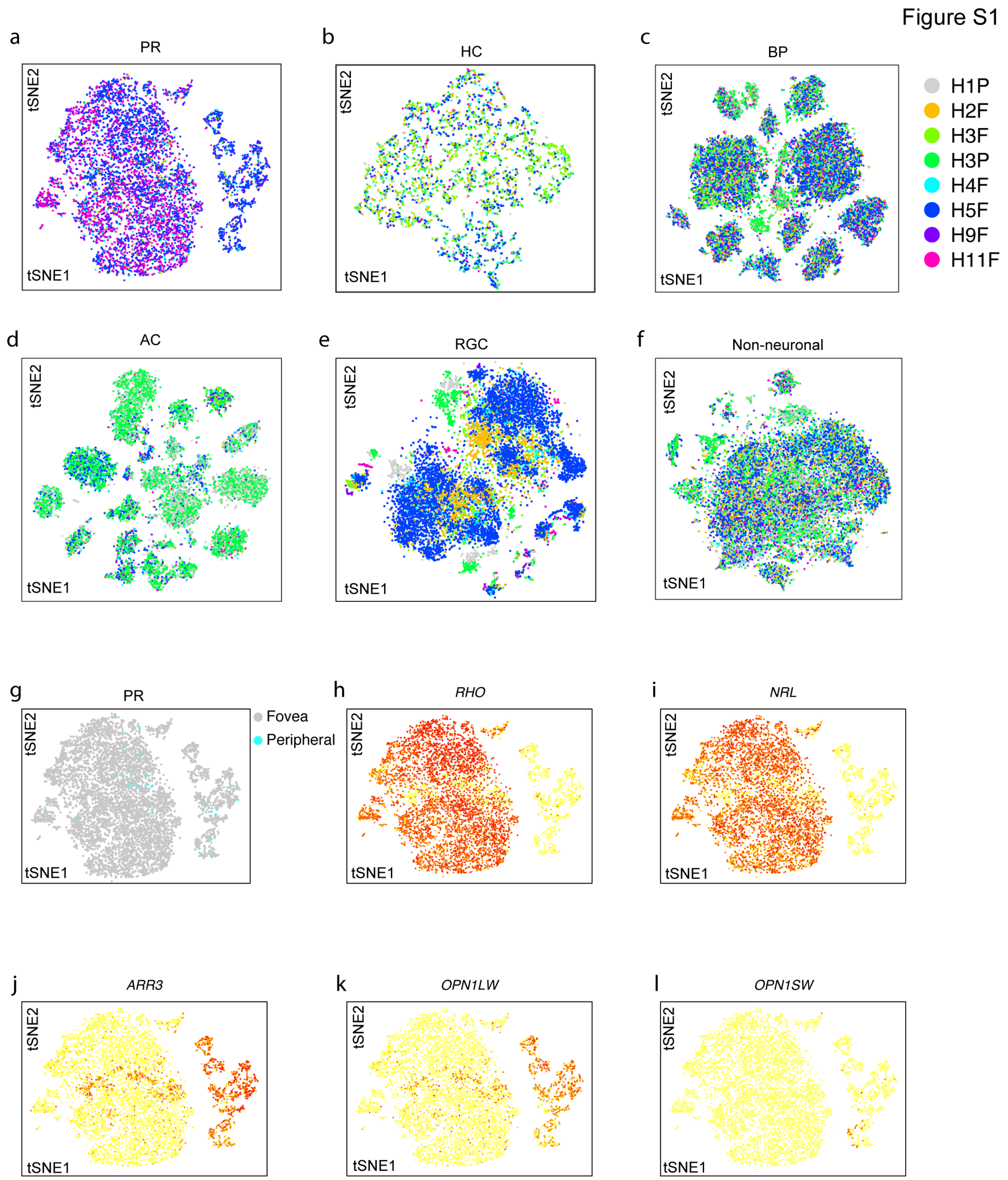
Figure S1
a
PR
b
HC
c
BP
H1P
H2F
H3F
H3P
H4F
H5F
H9F
H11F
d
AC
e
RGC
f
Non-neuronal
g
PR
Fovea
Peripheral
h
RHO
i
NRL
j
ARR3
k
OPN1LW
l
OPN1SW
tSNE1
tSNE2

Figure S2

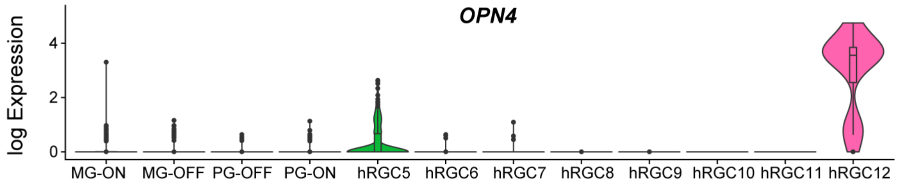

OPN4
log Expression
4
2
0
MG-ON
MG-OFF
PG-OFF
PG-ON
hRGC5
hRGC6
hRGC7
hRGC8
hRGC9
hRGC10
hRGC11
hRGC12

Figure S3

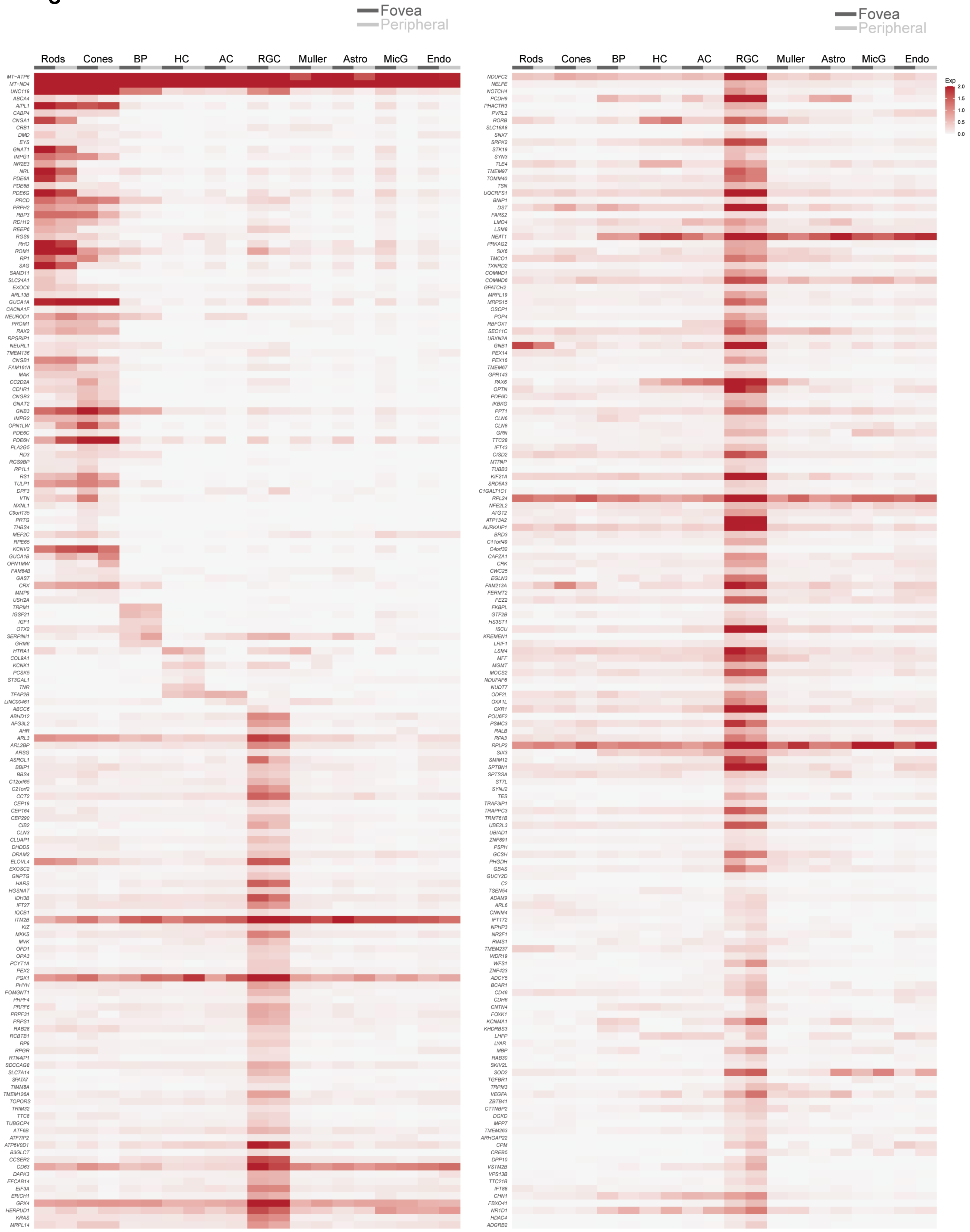

Fovea
Peripheral
Rods
Cones
BP
HC
AC
RGC
Muller
Astro
MicG
Endo
Exp
2.0
1.5
1.0
0.5
0.0
MT-ATP6
MT-ND4
UNC119
ABCA4
AIPL1
CABP4
CNGA1
CRB1
DMD
EYS
GNAT1
IMPG1
NR2E3
NRL
PDE6A
PDE6B
PDE6G
PRCD
PRPH2
RBP3
RDH12
REEP6
RGS9
RHO
ROM1
RP1
SAG
SAMD11
SLC24A1
EXOC6
ARL13B
GUCA1A
CACNA1F
NEUROD1
PROM1
RAX2
RPGRIP1
NEURL1
TMEM136
CNGB1
FAM161A
MAK
CC2D2A
CDHR1
CNGB3
GNAT2
GNB3
IMPG2
OPN1LW
PDE6C
PDE6H
PLA2G5
RD3
RGS9BP
RP1L1
RS1
TULP1
DPF3
VTN
NXNL1
C9orf135
PRTG
THBS4
MEF2C
RPE65
KCNV2
GUCA1B
OPN1MW
FAM84B
GAS7
CRX
MMP9
USH2A
TRPM1
IGSF21
IGF1
OTX2
SERPINI1
GRM6
HTRA1
COL9A1
KCNK1
PCSK5
ST3GAL1
TNR
TFAP2B
LINC00461
ABCC6
ABHD12
AFG3L2
AHR
ARL3
ARL2BP
ARSG
ASRGL1
BBIP1
BBS4
C12orf65
C21orf2
CCT2
CEP19
CEP164
CEP290
CIB2
CLN3
CLUAP1
DHDDS
DRAM2
ELOVL4
EXOSC2
GNPTG
HARS
HGSNAT
IDH3B
IFT27
IQCB1
ITM2B
KIZ
MKKS
MVK
OFD1
OPA3
PCYT1A
PEX2
PGK1
PHYH
POMGNT1
PRPF4
PRPF6
PRPF31
PRPS1
RAB28
RCBTB1
RP9
RPGR
RTN4IP1
SDCCAG8
SLC7A14
SPATA7
TIMM8A
TMEM126A
TOPORS
TRIM32
TTC8
TUBGCP4
ATF6B
ATF7IP2
ATP6V0D1
B3GLCT
CCSER2
CD63
DAPK3
EFCAB14
EIF3A
ERICH1
GPX4
HERPUD1
KRAS
MRPL14
NDUFC2
NELFE
NOTCH4
PCDH9
PHACTR3
PVRL2
RORB
SLC16A8
SNX7
SRPK2
STK19
SYN3
TLE4
TMEM97
TOMM40
TSN
UQCRFS1
BNIP1
DST
FARS2
LMO4
LSM8
NEAT1
PRKAG2
SIX6
TMCO1
TXNRD2
COMMD1
COMMD6
GPATCH2
MRPL19
MRPS15
OSCP1
POP4
RBFOX1
SEC11C
UBXN2A
GNB1
PEX14
PEX16
TMEM67
GPR143
PAX6
OPTN
PDE6D
IKBKG
PPT1
CLN6
CLN8
GRN
TTC28
IFT43
CISD2
MTPAP
TUBB3
KIF21A
SRD5A3
C1GALT1C1
RPL24
NFE2L2
ATG12
ATP13A2
AURKAIP1
BRD3
C11orf49
C4orf32
CAPZA1
CRK
CWC25
EGLN3
FAM213A
FERMT2
FEZ2
FKBPL
GTF2B
HS3ST1
ISCU
KREMEN1
LRIF1
LSM4
MFF
MGMT
MOCS2
NDUFAF6
NUDT7
ODF2L
OXA1L
OXR1
POU6F2
PSMC3
RALB
RPA3
RPLP2
SIX3
SMIM12
SPTBN1
SPTSSA
ST7L
SYNJ2
TES
TRAF3IP1
TRAPPC3
TRMT61B
UBE2L3
UBIAD1
ZNF891
PSPH
GCSH
PHGDH
GBAS
GUCY2D
C2
TSEN54
ADAM9
ARL6
CNNM4
IFT172
NPHP3
NR2F1
RIMS1
TMEM237
WDR19
WFS1
ZNF423
ADCY5
BCAR1
CD46
CDH6
CNTN4
FOXK1
KCNMA1
KHDRBS3
LHFP
LYAR
MBP
RAB30
SKIV2L
SOD2
TGFBR1
TRPM3
VEGFA
ZBTB41
CTTNBP2
DGKD
MPP7
TMEM263
ARHGAP22
CPM
CREB5
DPP10
VSTM2B
VPS13B
TTC21B
IFT88
CHN1
FBXO41
NR1D1
HDAC4
ADGRB2

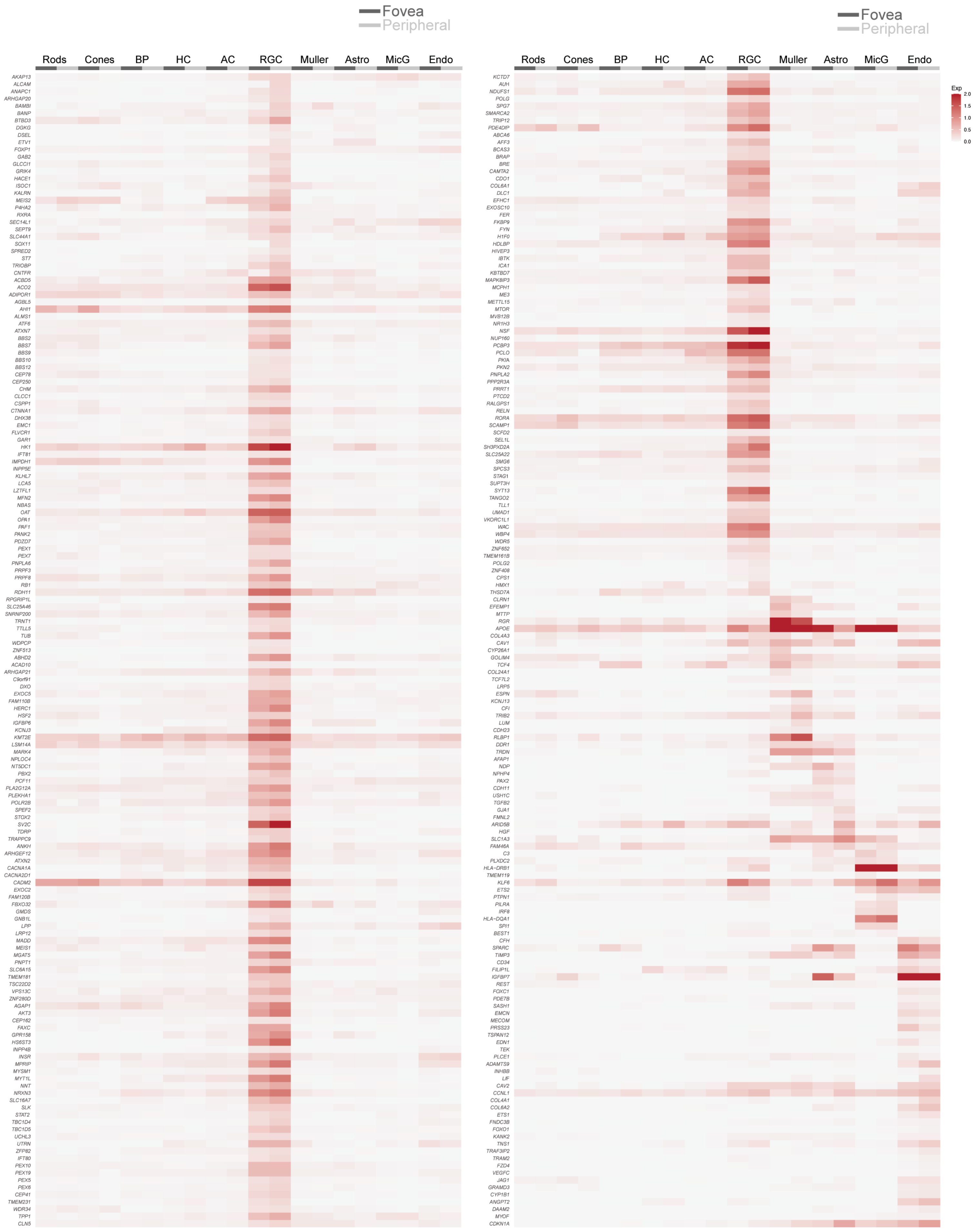

Fovea
Peripheral
Rods
Cones
BP
HC
AC
RGC
Muller
Astro
MicG
Endo
AKAP13
ALCAM
ANAPC1
ARHGAP20
BAMBI
BANP
BTBD3
DGKG
DSEL
ETV1
FOXP1
GAB2
GLCCI1
GRIK4
HACE1
ISOC1
KALRN
MEIS2
P4HA2
RXRA
SEC14L1
SEPT9
SLC44A1
SOX11
SPRED2
ST7
TRIOBP
CNTFR
ACBD5
ACO2
ADIPOR1
AGBL5
AHI1
ALMS1
ATF6
ATXN7
BBS2
BBS7
BBS9
BBS10
BBS12
CEP78
CEP250
CHM
CLCC1
CSPP1
CTNNA1
DHX38
EMC1
FLVCR1
GAR1
HK1
IFT81
IMPDH1
INPP5E
KLHL7
LCA5
LZTFL1
MFN2
NBAS
OAT
OPA1
PAF1
PANK2
PDZD7
PEX1
PEX7
PNPLA6
PRPF3
PRPF8
RB1
RDH11
RPGRIP1L
SLC25A46
SNRNP200
TRNT1
TTLL5
TUB
WDPCP
ZNF513
ABHD2
ACAD10
ARHGAP21
C9orf91
DXO
EXOC5
FAM110B
HERC1
HSF2
IGFBP6
KCNJ3
KMT2E
LSM14A
MARK4
NPLOC4
NT5DC1
PBX2
PCF11
PLA2G12A
PLEKHA1
POLR2B
SPEF2
STOX2
SV2C
TDRP
TRAPPC9
ANKH
ARHGEF12
ATXN2
CACNA1A
CACNA2D1
CADM2
EXOC2
FAM120B
FBXO32
GMDS
GNB1L
LPP
LRP12
MADD
MEIS1
MGAT5
PNPT1
SLC6A15
TMEM181
TSC22D2
VPS13C
ZNF280D
AGAP1
AKT3
CEP162
FAXC
GPR158
HS6ST3
INPP4B
INSR
MPRIP
MYSM1
MYT1L
NNT
NRXN3
SLC16A7
SLK
STAT2
TBC1D4
TBC1D5
UCHL3
UTRN
ZFP82
IFT80
PEX10
PEX19
PEX5
PEX6
CEP41
TMEM231
WDR34
TPP1
CLN5
Fovea
Peripheral
Rods
Cones
BP
HC
AC
RGC
Muller
Astro
MicG
Endo
Exp
2.0
1.5
1.0
0.5
0.0
KCTD7
AUH
NDUFS1
POLG
SPG7
SMARCA2
TRIP12
PDE4DIP
ABCA6
AFF3
BCAS3
BRAP
BRE
CAMTA2
CDO1
COL6A1
DLC1
EFHC1
EXOSC10
FER
FKBP9
FYN
H1F0
HDLBP
HIVEP3
IBTK
ICA1
KBTBD7
MAPK8IP3
MCPH1
ME3
METTL15
MTOR
MVB12B
NR1H3
NSF
NUP160
PCBP3
PCLO
PKIA
PKN2
PNPLA2
PPP2R3A
PRRT1
PTCD2
RALGPS1
RELN
RORA
SCAMP1
SCFD2
SEL1L
SH3PXD2A
SLC25A22
SMG6
SPCS3
STAG1
SUPT3H
SYT13
TANGO2
TLL1
UMAD1
VKORC1L1
WAC
WBP4
WDR5
ZNF652
TMEM161B
POLG2
ZNF408
CPS1
HMX1
THSD7A
CLRN1
EFEMP1
MTTP
RGR
APOE
COL4A3
CAV1
CYP26A1
GOLIM4
TCF4
COL24A1
TCF7L2
LRP5
ESPN
KCNJ13
CFI
TRIB2
LUM
CDH23
RLBP1
DDR1
TRDN
AFAP1
NDP
NPHP4
PAX2
CDH11
USH1C
TGFB2
GJA1
FMNL2
ARID5B
HGF
SLC1A3
FAM46A
C3
PLXDC2
HLA−DRB1
TMEM119
KLF6
ETS2
PTPN1
PILRA
IRF8
HLA−DQA1
SPI1
BEST1
CFH
SPARC
TIMP3
CD34
FILIP1L
IGFBP7
REST
FOXC1
PDE7B
SASH1
EMCN
MECOM
PRSS23
TSPAN12
EDN1
TEK
PLCE1
ADAMTS9
INHBB
LIF
CAV2
CCNL1
COL4A1
COL6A2
ETS1
FNDC3B
FOXO1
KANK2
TNS1
TRAF3IP2
TRAM2
FZD4
VEGFC
JAG1
GRAMD3
CYP1B1
ANGPT2
DAAM2
MYOF
CDKN1A

Table S1. Information of donors from whom retinas were obtained.

Donor ID	Retina region(s) sampled	Donor Age	Donor sex	COD	Duration to process after death (h)	10X Kit version
H1	Peripheral retina of left eye	74	Male	Lung Cancer	6	V2
H2	Fovea retina of both eyes	78	Male	Metastatic Melanoma to brain	14	V2
H3	Fovea and peripheral retina of right eye	60	Male	Left tonsillar squamous cell carcinoma metastatic to brain and left orbit	6.5	V2
H4	Fovea retina of left eye	64	Male	Diffuse B cell lymphoma spread to thorax and epigastrum	5	V2
H5	Fovea retina of left eye	69	Male	Metastatic Melanoma to brain, lung, spinal cord	3	V2
H9	Fovea retina of left eye	53	Female	Interstitial Lung Disease	5	V2
H11	Fovea retina of right eye	65	Male	Metastatic Melanoma	3	V3

Table S2. Publications on single cell RNA-seq profile of human retina.

Reference	Platform	Age	#Donors	# cells	Separate fovea/macula and periphery	# clusters	Identify types within classes	Cells or nuclei
[12]Peng et al., 2019	10X, V2	Adult	1	2,383	No	9	Yes	cells
[56]Hu et al., 2019	Modified STRT	Fetal week 5-24 weeks	19 embryos	2,421	No	21	No	cells
[55]Lukowski et al.,2019	10X, V2	Adult	3	20,009	No	17	Yes	cells
[59]Voigt et al., 2019	10X, V3	Adult	3	8,217	Yes	17	Yes	cryo-preserved cells
[58]Menon et al., 2019	10X, V3 and Seq-Well	Adult	6	23,432	Yes	9	No	cells
[57]Liang et al., 2019	ICELL8	Adult	3	5,873	Yes	7	No	nuclei
[60]Orozco et al., 2020	10X,V2,V3	Adult	5	100,055	Yes	46	Yes	Nuclei
[61]Sridhar et al., 2020	10X,V1,V2, V3	Fetal	4 embryos	61,164	Yes	10	No	Cells
This study	10X, V2 and V3	Adult	8	85,000	Yes	58	Yes	cells

Table S3. Sample quality matrix of each collection

Library ID	total cells passed initial 600 gene filter	Final number of cells	median_nGene	median_nTranscripts	median_nreads	mito_trans_ratio	Amacrine	Astrocytes	Bipolar	Cones	Endothelium	Horizontal	Microglia	Muller	RGC	Rods
**H1CD73dpS1**	6614	5883	1435	3085	10880	0.054	11.6	0.5	41.1	0.2	0.8	1.8	2	40.8	0.9	0.4
**H1CD90S1**	7049	6181	1523	3147	7634	0.052	51.3	0.9	7.1	0	0.1	0.2	0	31.9	8.5	0
**H2Fovea1S1**	2587	2191	985	1730	4662	0.055	2.5	0	35.3	1.6	0.4	5.1	0.2	25.9	27.8	1.2
**H2Fovea2S1**	4597	4202	960	1650.5	4526.5	0.035	1	0.1	31	0.8	0.4	4	0.8	40.8	20.6	0.4
**H3FoveaS1**	3838	3015	909	1749	7373	0.054	3.6	0.2	38.1	1.1	0.3	13.5	0.5	31.4	10.1	1.2
**H3FoveaS2**	4717	3565	934	1819	8498	0.054	3.2	0	41.9	1.2	0.3	14.2	0.4	27.5	9.2	2.1
**H3FoveaS3**	34	25	1394	2733	550895	0.036	12	0	52	0	0	12	0	20	4	0
**H3CD73dpS1**	4447	3955	1306	2772	17497	0.037	9.9	1.4	49.6	0.2	1.1	4.1	3	29.8	0.7	0.3
**H3CD73dpS2**	4203	3750	1270	2664	13471	0.037	10.6	1.3	47.9	0.2	1.2	3.6	2.8	31.3	0.8	0.4
**H3CD90S1**	5385	4879	1688	3480	13171	0.044	62.8	9.7	12	0	0.6	0.3	0	7.8	6.8	0
**H3CD90S2**	5144	4674	1642	3329.5	10239.5	0.043	63.8	9.3	12.3	0	0.8	0.3	0	7.4	6.2	0
**H4FoveaS1**	5961	4388	1306	2554	5635	0.059	11.8	0.2	27.5	0.9	0.5	5.2	0.4	33.7	11.7	8.1
**H5FoveaS1**	7998	6856	1342	2526	5212	0.041	5.9	0.2	29.8	4.4	0.3	2.8	0.4	22	26.3	7.9
**H5FoveaS2**	6426	5603	1323	2493	5151	0.042	6	0.3	30.5	5.1	0.3	2.8	0.4	21.1	24.1	9.4
**H5FoveaS3**	6411	5550	1293.5	2422	5044.5	0.043	5.3	0.2	31.4	4.9	0.3	3.1	0.5	19.9	25.1	9.4
**H5FoveaS4**	6524	5661	1313	2457	5223	0.042	6.2	0.2	30.6	4.8	0.4	3.2	0.3	20.7	24	9.6
**H5FoveaS5**	6385	5511	1331	2495	5768	0.042	5.4	0.2	31.5	5.3	0.2	3.2	0.4	22.2	21.7	10
**H9FoveaS1**	7943	5480	1220.5	2235.5	4399	0.053	2.5	0.2	27.4	6.3	0.5	1.3	1.3	25.5	3.3	31.6
**H11FoveaS1**	7798	4884	1395	2863	4062	0.185	5.2	0.4	35.4	0.5	0.3	1.3	2	6.9	4.9	43
